# SnRK1 activates autophagy via the TOR signaling pathway in *Arabidopsis thaliana*

**DOI:** 10.1371/journal.pone.0182591

**Published:** 2017-08-04

**Authors:** Junmarie Soto-Burgos, Diane C. Bassham

**Affiliations:** 1 Department of Genetics, Development and Cell Biology, Iowa State University, Ames, Iowa, United States of America; 2 Plant Sciences Institute, Iowa State University, Ames, Iowa, United States of America; University of Wisconsin Madison, UNITED STATES

## Abstract

Autophagy is a degradation process in which cells break down and recycle their cytoplasmic contents when subjected to environmental stress or during cellular remodeling. The *Arabidopsis thaliana* SnRK1 complex is a protein kinase that senses changes in energy levels and triggers downstream responses to enable survival. Its mammalian ortholog, AMPK, and yeast ortholog, Snf-1, activate autophagy in response to low energy conditions. We therefore hypothesized that SnRK1 may play a role in the regulation of autophagy in response to nutrient or energy deficiency in *Arabidopsis*. To test this hypothesis, we determined the effect of overexpression or knockout of the SnRK1 catalytic subunit KIN10 on autophagy activation by abiotic stresses, including nutrient deficiency, salt, osmotic, oxidative, and ER stress. While wild-type plants had low basal autophagy activity in control conditions, KIN10 overexpression lines had increased autophagy under these conditions, indicating activation of autophagy by SnRK1. A *kin10* mutant had a basal level of autophagy under control conditions similar to wild-type plants, but activation of autophagy by most abiotic stresses was blocked, indicating that SnRK1 is required for autophagy induction by a wide variety of stress conditions. In mammals, TOR is a negative regulator of autophagy, and AMPK acts to activate autophagy both upstream of TOR, by inhibiting its activity, and in a parallel pathway. Inhibition of *Arabidopsis* TOR leads to activation of autophagy; inhibition of SnRK1 did not block this activation. Furthermore, an increase in SnRK1 activity was unable to induce autophagy when TOR was also activated. These results demonstrate that SnRK1 acts upstream of TOR in the activation of autophagy in *Arabidopsis*.

## Introduction

Autophagy (self-eating) is a degradation process in which cells recycle cytoplasmic contents during development or when under stress. Upon autophagy activation, cellular components are degraded in the lysosome/vacuole and the products are recycled back into the cytoplasm [1]. Macroautophagy (hereafter referred to as autophagy) is characterized by the formation of a double-membrane vesicle called an autophagosome, which delivers cargo to the vacuole for degradation. To maintain homeostasis under normal conditions, cells have a basal level of autophagy to turn over damaged proteins or organelles, and the pathway is upregulated during stress [1]. Many autophagy-related (*ATG*) genes were initially identified in yeast [2–4], followed by identification of their homologs in other organisms. Major factors include ATG1, which together with ATG13 forms a complex involved in the induction of autophagy [5]. Two ubiquitin-like conjugates, ATG12/ATG5 and ATG8-PE, are recruited to the phagophore assembly site (PAS) and play an important role in autophagosome formation [5]. ATG9 is also recruited to the PAS and may function in the recruitment of other ATG components and membrane to the forming autophagosome [6]. In plants, autophagy is activated in response to many biotic and abiotic stresses, including pathogen attack [7, 8], nutrient deficiency [9, 10], salt, osmotic [11], endoplasmic reticulum (ER) [12], hypoxia [13] and oxidative stress [14].

A substantial number of upstream regulators of autophagy have been identified in animal cells, but the majority are not conserved in plants. One exception is the target of rapamycin (TOR), a phosphatidylinositol 3-kinase-related kinase that acts as a negative regulator of autophagy [15–17]. The central component of the TOR signaling pathway is the TOR complex, which in *Arabidopsis* consists of the serine/threonine kinase TOR itself [18], RAPTOR [19, 20], which presents substrates to TOR for phosphorylation [21], and LST8, which stabilizes the complex [22]. TOR coordinates autophagy with growth by controlling processes, for example translation initiation, that regulate growth in response to nutrient status [23, 24]. In nutrient-rich conditions, TOR is activated and inhibits autophagy, probably by phosphorylation of the ATG1 complex, which has been shown to regulate autophagy in *Arabidopsis* [25]. Upon nutrient deprivation, the TOR complex is inactivated, allowing the activation of autophagy and down-regulating growth. Consistent with this, an *Arabidopsis* knockout mutant in *TOR* is embryo-lethal [18], and lines with decreased *TOR* expression have reduced growth [23] and constitutive autophagy [17]. Conversely, TOR overexpression inhibits activation of autophagy in response to multiple abiotic stresses [26].

AMP-activated protein kinase (AMPK) in animals, and its yeast homolog sucrose non-fermenting 1 (Snf1), are positive regulators of autophagy. AMPK and Snf1 are energy and metabolic sensors that maintain cellular energy homeostasis [27, 28]. They are activated by an increase in the AMP:ATP and ADP:ATP ratios, which promotes phosphorylation of AMPK/Snf1 by upstream kinases [29–32]. Upon activation, AMPK implements an energy-saving program by transcriptional control and enzyme regulation [32]. Catabolic pathways such as fatty acid oxidation, glycolysis and autophagy are activated, while anabolic processes, including synthesis of cholesterol, proteins and fatty acids, are switched off [33]. AMPK/Snf1 regulates autophagy via two pathways: by inhibiting the TOR complex [34], therefore allowing autophagy to become active, or by the direct phosphorylation of ATG1, also leading to activation of autophagy [35–37].

The Snf1-related protein kinase 1 (SnRK1) is a plant ortholog of AMPK and Snf1, and is a heterotrimeric complex that functions as an energy sensor [33, 38]. SnRK1 is composed of a catalytic (α) and two regulatory (β, γ) subunits [33]. The regulatory subunits β and γ can be classified into two groups: the classical subunits [β_1_, β_2_ and γ] (conserved with mammals and yeast) and the plant-specific subunits [β_3_ and βγ] [32, 39, 40]. Even though the γ subunit exists in plants, the majority of SnRK1 active complexes contain the βγ hybrid subunit acting as the canonical γ subunit [41]. Three isoforms of the catalytic subunit exist in *Arabidopsis*, KIN10, KIN11 and KIN12, but only KIN10 and KIN11 appear to be expressed [42]. Of these two, KIN10 is responsible for most of the SnRK1 activity [43]. KIN10 and KIN11 can act in opposition to one another in some situations, indicating that they can perform separate functions. For example, overexpression of KIN10 results in late flowering while overexpression of KIN11 causes early flowering [44]. A *kin10 kin11* double knockout mutant is lethal, suggesting that there is also some redundancy in gene function [42]. A reduction in expression of *kin10* and *kin11* by virus-induced gene silencing leads to deformed leaves, inflorescences and flowers, short petioles, and reduced activation of stress and starvation genes, while single mutants of *kin10* and *kin11* resemble wild-type plants [42], indicating that SnRK1 functions in development and stress responses.

SnRK1 functions as an energy sensor, using carbohydrates as indicators of plant energy status [32]. For example, high concentrations of sugar phosphates, including trehalose-6-phosphate (T6P), can indicate energy availability and inhibit SnRK1 activity to maintain energy homeostasis [45–47]. The SnRK1 complex can activate basic leucine zipper (bZIP) transcription factors from the C and S family, including bZIP2, bZIP11 and bZIP63, in response to starvation [42, 48, 49]. This in turn leads to upregulation of the expression of genes of various catabolic pathways, including autophagy and degradation of cell wall components, starch, amino acids, sucrose, lipids and protein, providing alternative sources of energy and metabolites [42]. This suggests that SnRK1 has a regulatory influence on global plant metabolism, growth and energy balance [42].

In addition to inhibition of SnRK1 by T6P, type 2C protein phosphatases (PP2C) can also negatively regulate SnRK1 by dephosphorylation [50], leading to its inactivation. Abscisic acid (ABA) inhibits PP2C and therefore can positively regulate SnRK1 by allowing its activation [50]. In contrast with AMPK, SnRK1 has not been shown to be allosterically regulated by AMP, but AMP instead affects its rate of dephosphorylation and therefore activity [38, 51].

Recent studies have shown that *Arabidopsis* KIN10 interacts with the TOR complex subunit RAPTOR *in vivo* and can phosphorylate RAPTOR *in vitro*, as for its mammalian orthologs [52]. This suggests a crosstalk between these two regulators of energy metabolism and growth. Since the yeast and mammalian orthologs of SnRK1 are positive regulators of autophagy [37], and AMPK can act either through the TOR signaling pathway or independently of it, we hypothesized that KIN10 functions in the regulation of autophagy in plants, potentially through TOR. Our results demonstrate that KIN10 is an activator of autophagy in *Arabidopsis* and that it acts upstream of TOR in regulation of autophagy.

## Results

### Overexpression of KIN10 leads to increased autophagy

**Fig 1:**
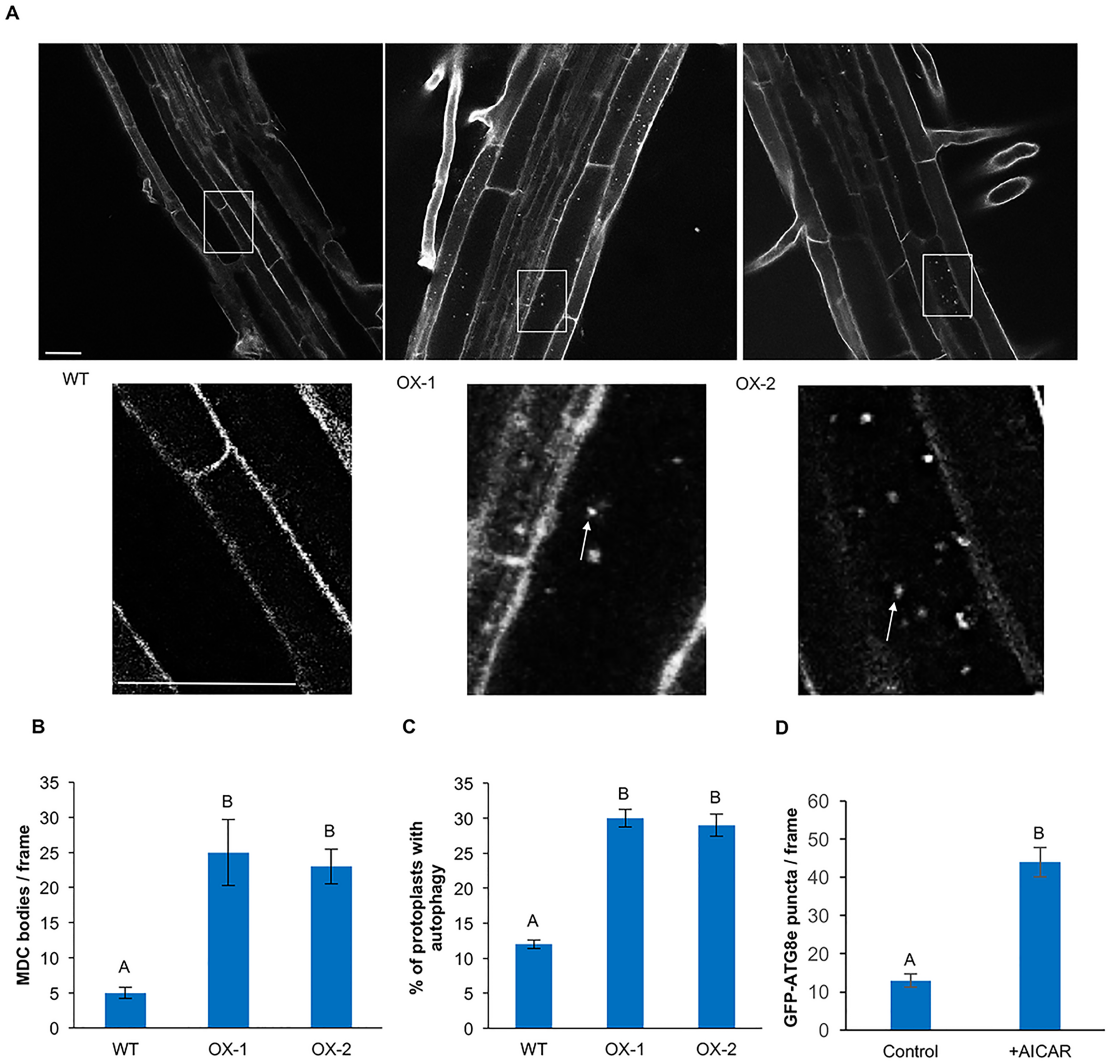
Overexpression of KIN10 leads to increased basal autophagy. (A) WT, OX-1 and OX-2 seedlings were grown on standard growth medium for 7 days, then stained with MDC. Confocal microscopy was used to visualize autophagosomes in roots. The insets show enlargements of the indicated boxes. Arrows indicate MDC-labeled structures. Scale bars = 20 μm. (B) Quantification of the number of autophagosomes in seedlings grown as in (A). KIN10 overexpression lines have increased autophagy activity when compared to WT. Different letters denote statistical significance for three biological replicates with at least 10 images per replicate, p<0.05, t-test. Error bars indicate standard error. (C) The autophagosome marker GFP-ATG8e was transiently expressed in leaf protoplasts from the indicated genotypes and the percentage of protoplasts with active autophagy determined. A protoplast was considered to have active autophagy if it contained 3 or more GFP-ATG8e-labeled autophagosomes. KIN10 overexpression lines have increased autophagy activity when compared to WT. Different letters denote statistical significance for three biological replicates, with 100 protoplasts per sample per replicate, p<0.05, t-test. Error bars indicate standard error. (D) Seven-day-old GFP-ATG8e-expressing seedlings were transferred to liquid medium plus or minus 10 mM AICAR for 1 hour, and the number of autophagosomes per unit area counted. Seedlings treated with AICAR had higher autophagy activity than the control. Different letters denote statistical significance for three biological replicates with at least 10 images per replicate, p<0.05, t-test. Error bars indicate standard error.

AMPK and Snf1, the mammalian and yeast orthologs of SnRK1, have been demonstrated to be positive regulators of autophagy [35–37]. To determine whether SnRK1 can regulate autophagy in *Arabidopsis*, autophagy activity was assessed in the previously described *KIN10* overexpression lines OX-1 and OX-2 [42]. Seven-day-old WT, OX-1 and OX-2 seedlings grown under standard growth conditions were stained with the acidotropic dye monodansycadaverine (MDC) [53] and visualized by confocal microscopy (Fig 1A). We have previously shown that under our conditions, MDC labeling in roots co-localizes with labeling by the specific autophagosome marker GFP-ATG8e, indicating that the fluorescent structures correspond to autophagosomes [54]. While WT seedlings had very few visible autophagosomes, autophagosomes were abundant in both overexpression lines. Autophagy was quantified by counting autophagosomes in epifluorescence images of equal area of the elongation zone of the roots for each genotype (Fig 1B). While WT plants had the expected low basal autophagy activity, the KIN10 overexpression lines had significantly increased autophagy activity when compared to WT. There was no significant difference in autophagy activity between OX-1 and OX-2 (Fig 1B).

To confirm these results, protoplasts were prepared from leaves of 4–6 week-old WT, OX-1 and OX-2 *Arabidopsis* plants, followed by transient expression of GFP-ATG8e to label autophagosomes [53]. The protoplasts were imaged by confocal (S1A Fig) and fluorescence microscopy, and the percentage of protoplasts with active autophagy was determined (Fig 1C). A protoplast was considered to have activated autophagy if it contained 3 or more GFP-labeled autophagosomes or autophagic bodies [55]. Consistent with the results from MDC staining, a low percentage of protoplasts (12%) from WT plants had active autophagy, while the percentage of protoplasts from KIN10 overexpression lines with active autophagy was significantly higher (30%). There was no difference in the percentage of protoplasts with autophagy between KIN10 overexpression lines (Fig 1C). All genotypes expressed similar levels of GFP-ATG8e when compared to the loading control (S1B Fig).

As an alternative approach to increasing SnRK1 activity, seedlings were incubated with 5-aminoimidazole-4-carboxamide ribonucleoside monophosphate (AICAR), a chemical activator of AMPK-like kinases [56]. GFP-ATG8e-expressing [14] seedlings were grown under standard conditions on half-strength MS for 7 days and then transferred to the same medium supplemented with 10 mM AICAR for 1 hour. In the presence of AICAR, autophagy activity was significantly increased when compared to the control (Fig 1D). Together, these results suggest that KIN10 is a positive regulator of autophagy in *Arabidopsis* and that an increase in KIN10 activity either by overexpression or by chemical activation leads to increased basal autophagy.

### Autophagy is blocked during abiotic stress in a *kin10* mutant

**Fig 2:**
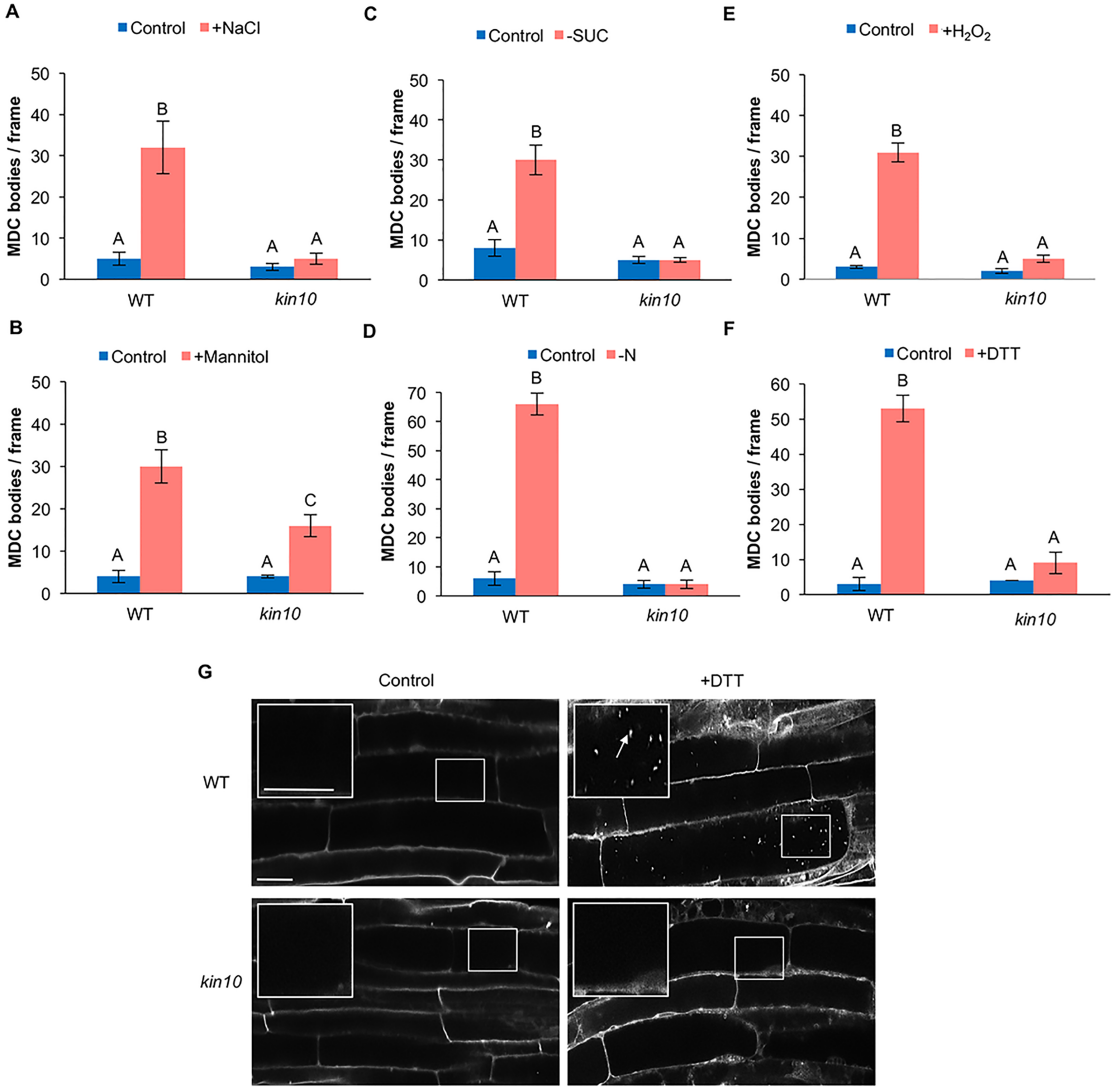
Autophagy is blocked during abiotic stress in *kin10* mutant seedlings. Seven-day-old WT and *kin10* seedlings were transferred to ½ MS liquid medium supplemented with 160 mM NaCl for 6 hours (A), ½ MS liquid medium supplemented with 350 mM mannitol for 6 hours (B), ½ MS plates lacking sucrose for 4 days in the dark (C), ½ MS plates lacking nitrogen for 4 days (D), ½ MS liquid medium supplemented with 10 mM hydrogen peroxide for 2 hours (E), or ½ MS liquid medium supplemented with 2 mM DTT (ER stress) for 6 hours (F). Seedlings were stained with MDC and autophagosomes counted. Autophagy was activated in WT seedlings after abiotic stress, while in *kin10* mutant seedlings autophagy was not induced in most conditions. The exception was osmotic stress, in which activation of autophagy in the *kin10* mutant was reduced but not completely blocked. Different letters denote statistical significance, p<0.05, t-test. Error bars indicate standard error. (G) Confocal images of WT and *kin10* mutant roots under control conditions and ER stress as a representative stress. The insets show enlargements of the indicated boxes. White arrows point to autophagosomes. Scale bars = 20 μm.

Since overexpression of KIN10 caused constitutive activation of autophagy, we hypothesized that loss of function of KIN10 would have a negative effect on the activation of autophagy. To test this hypothesis, a previously described knockout mutant with a T-DNA insertion in the eleventh exon of the *KIN10* gene [57, 58] was obtained from the Arabidopsis Biological Resource Center. WT and *kin10* mutant seedlings were grown on half-strength MS plates for seven days, followed by incubation under abiotic stress conditions (salt, osmotic, oxidative, endoplasmic reticulum (ER) stresses or fixed-carbon or nitrogen deficiency) to activate autophagy. Autophagy was detected by MDC staining and quantified by counting the number of autophagosomes per unit area. Under normal conditions, the *kin10* mutant had a low basal level of autophagy, similar to WT seedlings (Fig 2). As expected, autophagy was induced in WT seedlings under salt (Fig 2A), osmotic (Fig 2B), starvation (Fig 2C and 2D), oxidative (Fig 2E), and ER stress (Fig 2F–2G). In the *kin10* mutant, activation of autophagy by stress was completely blocked (Fig 2A and 2C–2F), with the exception of osmotic stress. Upon osmotic stress treatment, activation of autophagy could be observed in the *kin10* mutant, although the degree of activation was significantly reduced when compared to WT (Fig 2B). Confocal images of MDC-stained WT and *kin10* seedlings in control and ER stress conditions are shown as an example (Fig 2G).

**Fig 3:**
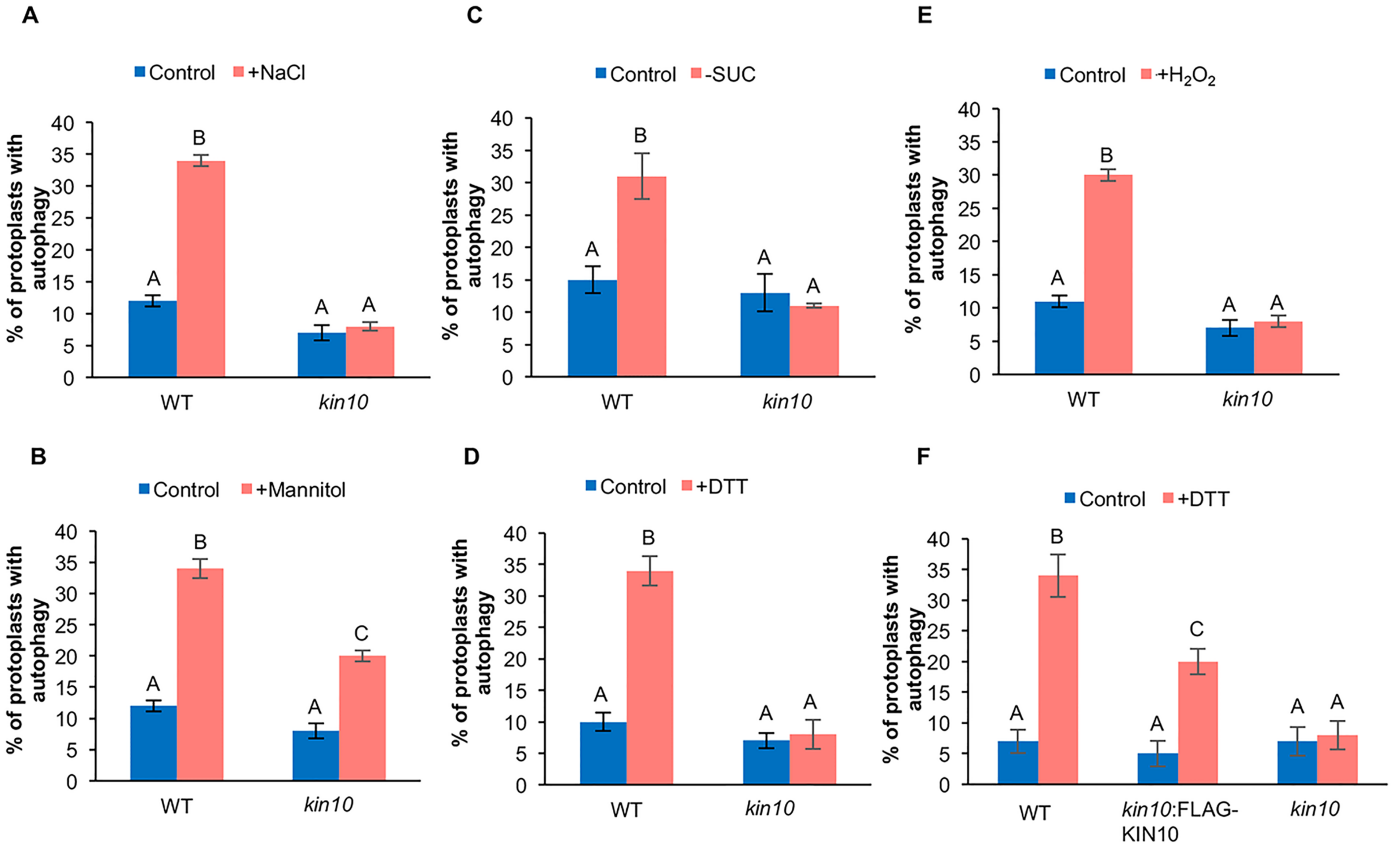
Autophagy is blocked during abiotic stress in *kin10* mutant protoplasts. WT and *kin10* protoplasts were transiently transformed with the autophagy marker GFP-ATG8e, incubated overnight to allow expression, and then the protoplast solution was supplemented with 160 mM NaCl for 6 hours (A), supplemented with 350 mM mannitol for 6 hours (B), incubated plus or minus 1% sucrose for 48 hours (C), supplemented with 2 mM DTT for 6 hours (D), or supplemented with 10 mM hydrogen peroxide for 2 hours (E). Autophagosomes were visualized by epifluorescence microscopy and the percentage of protoplasts with active autophagy determined. Different letters denote statistical significance for three biological replicates with 100 protoplasts for each sample per replicate, p<0.05, t-test. Error bars indicate standard error. Autophagy was activated in WT protoplasts after abiotic stress, but not in *kin10* mutant protoplasts. Upon osmotic stress, activation of autophagy in the *kin10* mutant was reduced but not completely blocked. (F) Protoplasts were co-transformed with FLAG-KIN10 and GFP-ATG8e constructs to confirm that the lack of autophagy in *kin10* was due to disruption of the *KIN10* gene. DTT was used to induce autophagy as in (D). Expression of FLAG-KIN10 restored the induction of autophagy during ER stress in the *kin10* mutant. Different letters denote statistical significance for three biological replicates with 100 protoplasts for each sample per replicate, p<0.05, t-test. Error bars indicate standard error.

To confirm that the loss of *kin10* prevents activation of autophagy under most abiotic stress conditions, protoplasts were prepared from leaves of 4–6 week old WT and *kin10 Arabidopsis* plants, followed by transient expression of GFP-ATG8e to label autophagosomes. Protoplasts were incubated under abiotic stress conditions, followed by quantification of the percentage of protoplasts with active autophagy (Fig 3A–3E, S2 Fig). Consistent with the MDC staining, activation of autophagy by abiotic stress was observed in protoplasts from WT plants, while those from the *kin10* mutant lacked induction of autophagy under salt (Fig 3A), starvation (Fig 3C), ER (Fig 3D) and oxidative stress (Fig 3E). After osmotic stress, autophagy was induced in both WT and *kin10* mutant protoplasts, although in the *kin10* mutant plants the level of autophagy activity was significantly lower than in WT plants (Fig 3B). Confocal images of WT and *kin10* protoplasts in control and ER stress conditions are shown as an example (S2A Fig), and both genotypes expressed GFP-ATG8e to similar levels when compared to the loading control (S2B Fig).

Finally, we confirmed that the autophagy defects observed in the *kin10* mutant were caused by loss of function of the *KIN10* gene. N-terminally FLAG-tagged KIN10 was co-expressed with GFP-ATG8e in leaf protoplasts from *kin10* plants and subjected to ER stress as a representative stress condition. Confocal images of WT, *kin10* and *kin10*:FLAG-KIN10 protoplasts in control and ER stress conditions are shown in S3A Fig, and all genotypes expressed GFP-ATG8e to similar levels compared to the loading control (S3B Fig). Unlike the *kin10* mutant expressing GFP-ATG8e alone, autophagy was activated in the FLAG-KIN10-expressing *kin10* protoplasts, indicating that the *KIN10* transgene was able to complement the autophagy phenotype of the *kin10* mutant (Figs 3F and S3A). The degree of autophagy activation in the complemented protoplasts was still significantly lower than that of WT protoplasts, possibly due to differences in expression level. Our results indicate that KIN10 is required for activation of autophagy by abiotic stress, but not for the basal autophagy observed under control conditions.

### Inhibition of the SnRK1 complex blocks activation of autophagy by abiotic stress

**Fig 4:**
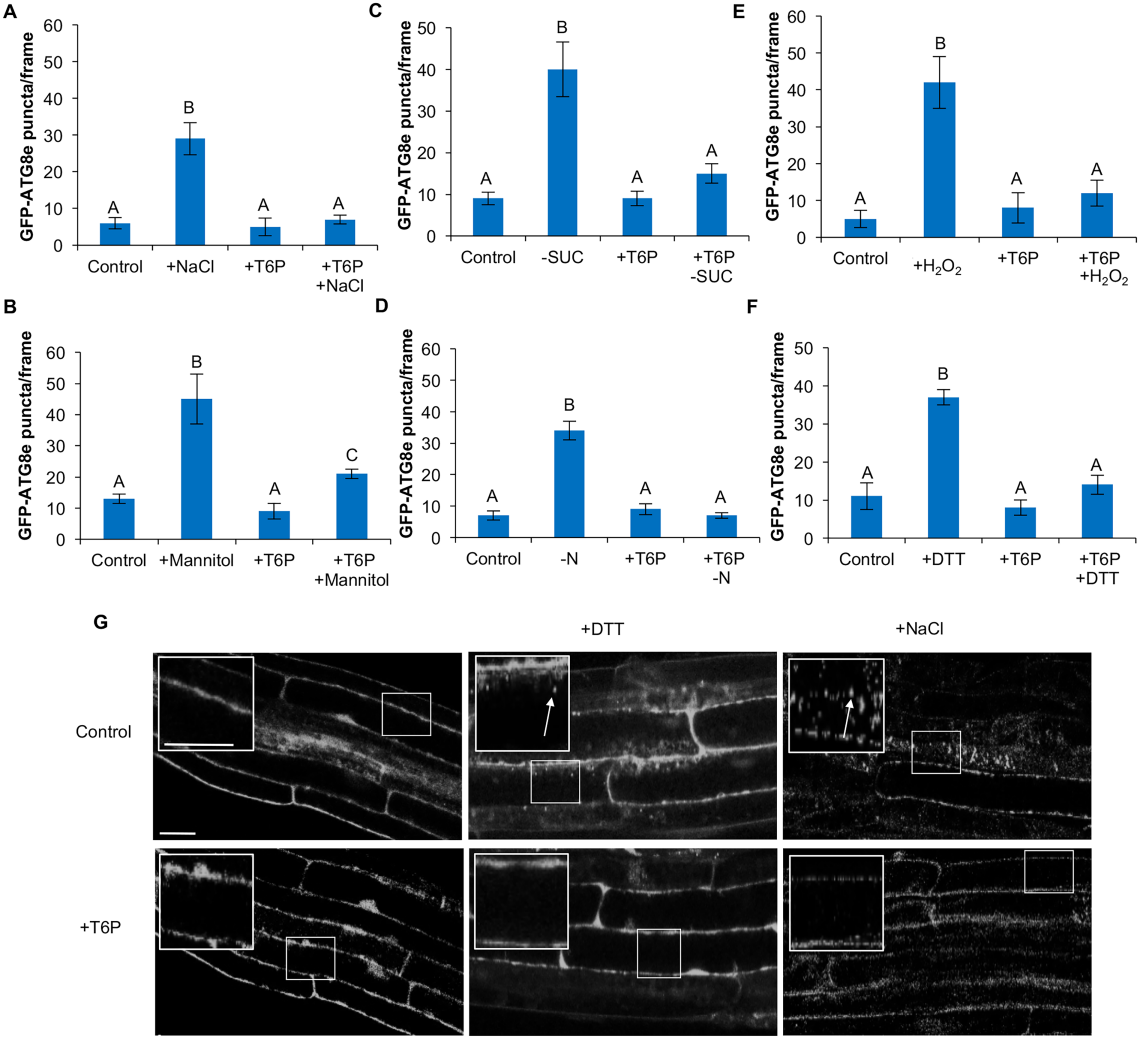
Inhibition of SnRK1 activity by T6P inhibits autophagy under abiotic stress. Seven-day-old GFP-ATG8e seedlings were transferred to ½ MS liquid medium supplemented with 0.1 mM T6P for 3 hours as control, or liquid medium supplemented with 160 mM NaCl for 6 hours and 0.1 mM T6P for the last 3 hours of treatment (A), liquid medium supplemented with 350 mM mannitol for 6 hours and 0.1 mM T6P for the last 3 hours of treatment (B), ½ MS plates lacking sucrose for 4 days in the dark followed by 0.1 mM T6P treatment in liquid medium for 3 hours (C), ½ MS plates lacking nitrogen for 4 days followed by 0.1 mM T6P treatment in liquid medium for 3 hours (D), liquid medium supplemented with 0.1 mM T6P for 3 hours and 10 mM hydrogen peroxide added for the last 2 hours (E), or liquid medium supplemented with 2 mM DTT for 6 hours and 0.1 mM T6P for the last 3 hours of treatment (F). Autophagosomes were imaged using epifluorescence microscopy and counted. Addition of T6P blocked the activation of autophagy in most conditions. In osmotic stress, autophagy was reduced but not completely blocked by T6P. Different letters denote statistical significance for three biological replicates with at least 10 frames per replicate, p<0.05, t-test. Error bars indicate standard error. (G) Confocal images of roots of GFP-ATG8e-expressing seedlings under control conditions, ER stress and salt stress as representative stresses. The insets show enlargements of the indicated boxes. White arrows point to autophagosomes. Scale bars = 20 μm.

Trehalose-6-phosphate (T6P) is a sugar synthesized from UDP-glucose and glucose-6-phosphate by trehalose-6-phosphate synthase [59] and has been shown to inhibit the activity of the SnRK1 complex [45]. To confirm that loss of KIN10 activity prevents activation of autophagy during stress, 7-day-old GFP-ATG8e-expressing seedlings were subjected to abiotic stress treatments as described above and co-treated with 0.1 mM T6P for the last 3 hours of the stress treatment (Fig 4). Upon exposure to salt, starvation, oxidative or ER stresses, treatment with T6P prevented activation of autophagy (Fig 4A and 4C–4F). Consistent with the *kin10* mutant phenotype, treatment with T6P did not completely inhibit the induction of autophagy during osmotic stress, although autophagy activity was significantly reduced when compared to the untreated control (Fig 4B). As examples, confocal images upon ER and salt stress are shown (Fig 4G). These results demonstrate that the activity of the SnRK1 complex is necessary for the induction of autophagy in response to abiotic stress, and SnRK1 is therefore a positive regulator of autophagy.

### KIN10 acts upstream of TOR in regulation of autophagy

The mammalian ortholog of KIN10, AMPK, can regulate autophagy through the mTOR signaling pathway or through an mTOR-independent pathway [35, 36]. In plants, TOR has been identified as a negative regulator of autophagy [17], and the TOR complex subunit RAPTOR can be phosphorylated by SnRK1 [52], but the relationship between these components in regulation of autophagy is unknown. We hypothesized that KIN10 acts upstream of TOR in the regulation of autophagy. If our hypothesis is true, then (a) blocking both TOR activity and KIN10 activity will lead to constitutive autophagy and (b) activating both TOR and KIN10 will result in a block in autophagy. We tested this hypothesis using genetic and chemical approaches to inhibit or activate TOR and KIN10.

#### Disruption of TOR and KIN10 activity leads to constitutive autophagy

**Fig 5:**
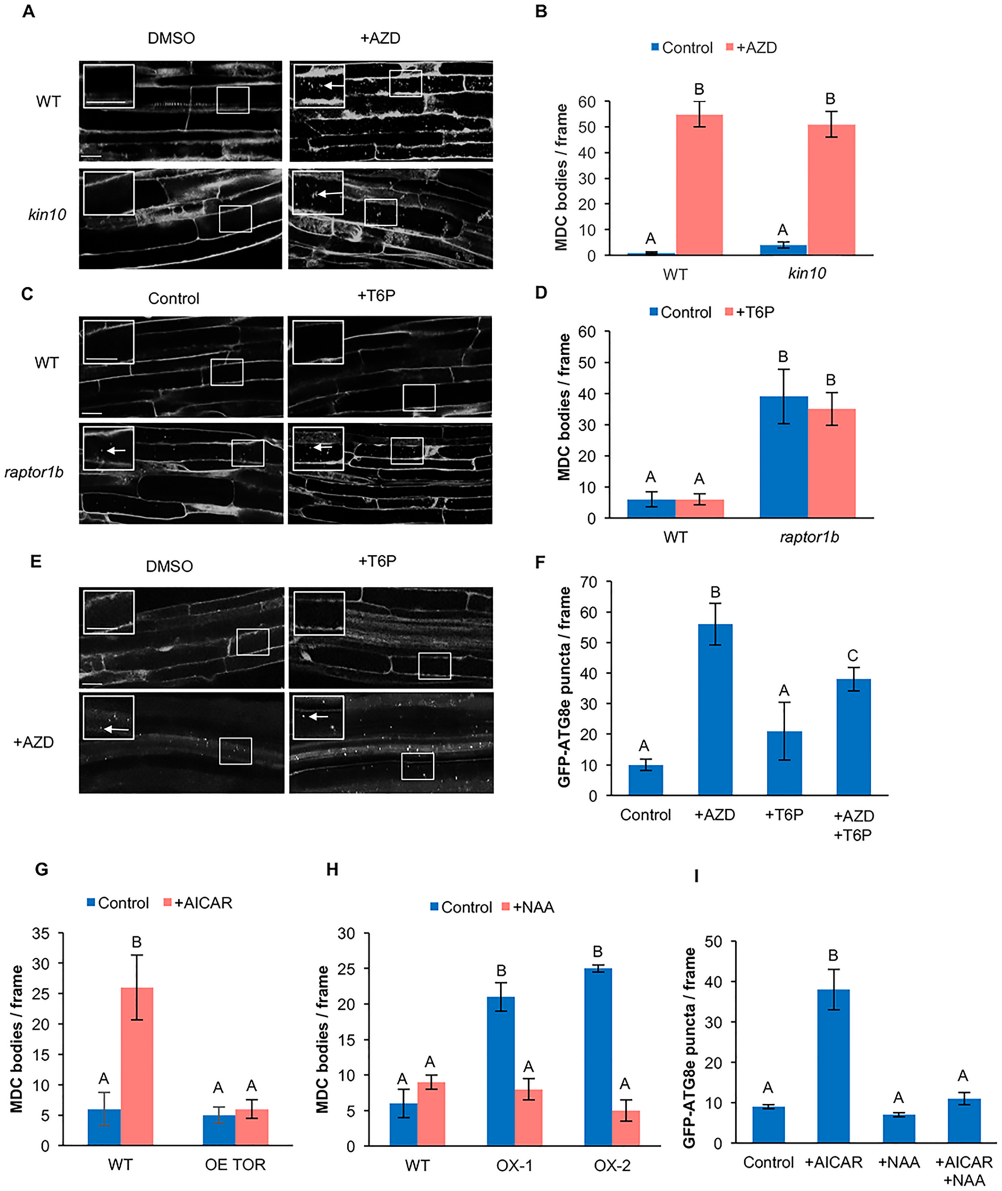
SnRK1 acts upstream of TOR in the autophagy pathway. (A) WT and *kin10* seedlings were grown on ½ MS plates for 7 days. Seedlings were transferred to ½ MS liquid medium supplemented with 10 μM AZD or DMSO for 3 hours, followed by MDC staining. Confocal microscopy was used to visualize autophagosomes (white arrows) in roots. The insets show enlargements of the indicated boxes. Scale bars = 20 μm. (B) Quantification of autophagy activity as shown in (A). Upon inhibition of TOR with AZD, autophagy was still activated in *kin10* mutant seedlings. (C) WT and *raptor1b* seedlings were grown on ½ MS plates for 7 days. Seedlings were transferred to ½ MS liquid medium supplemented with 0.1 mM T6P for 3 hours, followed by MDC staining. Confocal microscopy was used to visualize autophagosomes (white arrows) in roots. The insets show enlargements of the indicated boxes. Scale bars = 20 μm. (D) Quantification of autophagy activity in (C). Upon inhibition of SnRK1 with T6P, autophagy activity was not affected in *raptor1b* seedlings. (E) GFP-ATG8e seedlings were grown on ½ MS plates for 7 days. Seedlings were transferred to ½ MS liquid medium supplemented with 0.1 mM T6P or 10 μM AZD or T6P plus AZD for 3 hours. Confocal microscopy was used to visualize autophagosomes (white arrows) in roots. The insets show enlargements of the indicated boxes. Scale bars = 20 μm (F) Quantification of autophagosomes labeled with GFP-ATG8e in (E). Upon inhibition of both TOR and SnRK1, autophagy was activated. (G) WT and OE TOR seedlings were grown on ½ MS plates for 7 days. Seedlings were transferred to ½ MS liquid medium supplemented with 10 mM AICAR for 1 hour, followed by MDC staining, and autophagosomes counted. Overexpression of TOR was able to suppress AICAR-induced autophagy. (H) WT, KIN10 OX-1 and KIN10 OX-2 seedlings were grown on ½ MS plates for 7 days. Seedlings were transferred to ½ MS liquid medium supplemented with 20 mM NAA or DMSO for 6 hours, stained with MDC and autophagosomes counted. Activation of TOR by auxin inhibited the constitutive autophagy in KIN10 overexpression lines. (I) Seven-day-old GFP-ATG8e seedlings were transferred to ½ MS liquid medium supplemented with 10 mM AICAR or 20 nM NAA or both AICAR and NAA. Activation of TOR by NAA blocked induction of autophagy by AICAR. For all graphs, different letters denote statistical significance for three biological replicates with at least 10 frames per replicate, p<0.05, t-test. Error bars indicate standard error.

Previous studies have shown that the chemical AZD8055 (AZD) inhibits TOR kinase activity in *Arabidopsi*s [60] and activates autophagy [26]. Decreased TOR activity leads to constitutive autophagy [17], while a *kin10* mutant is unable to activate autophagy upon abiotic stress (Fig 2). Seven-day-old WT and *kin10* seedlings were transferred to liquid medium supplemented with 10 μM AZD for 3 hours [60], followed by MDC staining. After AZD treatment, autophagy was active in WT seedlings, consistent with the role of TOR as a negative regulator of autophagy [26]. Upon inhibition of TOR, autophagy was still activated in *kin10* mutant seedlings (Fig 5A and 5B).

The TOR complex is composed of the TOR kinase catalytic subunit, RAPTOR and LST8, in which RAPTOR delivers the substrate to the TOR kinase. In *Arabidopsis*, two *RAPTOR* genes have been identified, *RAPTOR1A* and *RAPTOR1B* [19], and disruption of *RAPTOR1B* results in the inactivation of the TOR complex [26]. Seven-day-old WT and *raptor1b* seedlings were transferred to liquid medium supplemented with 0.1 mM T6P for 3 hours, followed by MDC staining. Under control conditions *raptor1b* seedlings have constitutive autophagy when compared to WT, consistent with disruption of TOR activity. Upon inhibition of SnRK1 by T6P, autophagy activity in the *raptor1b* mutant was not affected (Fig 5C and 5D).

To confirm these genetic results using chemical inhibition of TOR and KIN10, 7-day-old GFP-ATG8e seedlings were transferred to liquid medium and treated with AZD, T6P or co-treated with AZD and T6P for 3 hours. Under control conditions autophagy was at a low basal level. Upon inhibition of TOR by AZD, autophagy was activated. Autophagy activity was not affected in seedlings treated with T6P. Upon co-treatment with AZD and T6P, autophagy activity was induced (Fig 5E and 5F). These data are all consistent with our hypothesis that KIN10 is upstream of TOR in the regulation of autophagy.

#### Activation of both TOR and KIN10 blocks autophagy

As a second approach to test our hypothesis that KIN10 is upstream of TOR for activating autophagy, the effect of activation of SnRK1 on seedlings with increased TOR activity was assessed. Seven-day-old WT and TOR overexpressing (OE TOR) [61] seedlings were transferred to liquid medium supplemented with 10 mM AICAR (SnRK1 activator) for 1 hour, followed by MDC staining. Under control conditions the autophagy activity in WT and OE TOR seedlings was at a low basal level. After AICAR treatment, WT seedlings have induced autophagy activity. Upon activation of SnRK1 by AICAR, seedlings overexpressing TOR are unable to activate autophagy (Fig 5G).

Recent studies have shown that the phytohormone auxin can activate TOR kinase [62] and therefore inhibit autophagy [26]. Seven-day-old WT and KIN10 OX-1 and OX-2 seedlings were transferred to liquid medium supplemented with 20 nM NAA for 6 hours, followed by MDC staining. Under control conditions the autophagy activity in WT seedlings was at a low basal level, while KIN10 overexpression seedlings have constitutive autophagy. After NAA treatment, the autophagy activity in KIN10 overexpression lines was significantly reduced (Fig 5H).

Finally, 7-day-old GFP-ATG8e seedlings were transferred to liquid medium and treated with NAA for 6 hours, AICAR for 1 hour, or co-treated with NAA for 6 hours plus AICAR for the last hour of incubation. Under control conditions or upon treatment with NAA, autophagy was at a low basal level. Upon activation of SnRK1 by AICAR, autophagy was activated. Upon co-treatment with AICAR and NAA, induction of autophagy by AICAR was blocked (Fig 5I).

In summary, activation of autophagy by increasing KIN10 activity is blocked upon activation of TOR, whereas disruption of KIN10 activity does not block the constitutive autophagy seen upon inhibition of TOR. Taken together, these results demonstrate that KIN10 acts upstream of TOR in the regulation of autophagy.

## Discussion

Autophagy is a vacuolar degradation pathway induced by multiple environmental stresses in plants, including nutrient starvation, osmotic, oxidative and ER stress, and during certain stages of development such as senescence [1]. Regulation of autophagy has been widely studied in animals and yeast, but is still poorly understood in plants. A few regulators of autophagy have been identified in plants, such as the ATG1/ATG13 complex, which activates autophagy in response to nutrient stress [25], IRE1b, which functions in response to ER stress [12], PTEN, which regulates autophagy in pollen tubes [63], and the TOR complex, a negative regulator of autophagy under nutrient-rich conditions [17]. In this paper, we demonstrate that the SnRK1 complex catalytic subunit KIN10 is a positive regulator of autophagy and that it functions upstream of the TOR complex in the activation of autophagy.

The SnRK1 complex acts as an energy sensor and is activated under conditions of low energy or metabolic stress to inhibit growth and conserve energy [64]. SnRK1 regulates metabolism by the phosphorylation and inactivation of important plant metabolic enzymes, including 3-hydroxymethyl-3-methylglutaryl-CoA reductase [65, 66], sucrose phosphate synthase, nitrate reductase [67] and trehalose phosphate synthase 5 [68]. SnRK1 also indirectly controls carbohydrate metabolism by modulating the transcription of genes such as sucrose synthase, involved in sucrose degradation, and α-amylase, involved in starch degradation [69]. In response to low energy conditions, SnRK1 mammalian and yeast orthologs can activate autophagy via inactivation of the TOR complex [37]. We found that the overexpression of the *KIN10* gene results in constitutive activation of the autophagy pathway in *Arabidopsis*. Furthermore, addition of the AMPK activator AICAR [56] to seedlings to activate the SnRK1 complex led to induction of autophagy. The activation of SnRK1 results in the upregulation of catabolism and downregulation of anabolism [59], and autophagy is potentially one of the mechanisms used to maintain energy balance. In low energy conditions, autophagy can recycle cytoplasmic components, producing both raw materials that can be used in biosynthetic pathways when substrates are limiting, and alternative TCA cycle substrates for ATP production, thus helping to maintain homeostasis.

Analysis of a *kin10* knockout mutant revealed that KIN10 is necessary to activate autophagy in response to nutrient starvation, salt stress, oxidative stress and ER stress. We hypothesize that upon nutrient deprivation SnRK1 is activated by low energy and in turn activates autophagy to compensate for the nutrient deficiency, thus contributing to stress tolerance. SnRK1 has also been linked to other plant stress responses, including salt tolerance [70] and pathogen resistance [71, 72]. During salt stress, abscisic acid (ABA) levels are increased [73], which potentially can lead to the activation of the SnRK1 complex [50], therefore promoting autophagy. ABA signaling is also critical for osmotic stress responses, which are activated either through ABA-dependent or ABA-independent but DREB2-dependent pathways [74]. During osmotic stress, activation of autophagy in the *kin10* mutant was only partially blocked, indicating that an alternative pathway may exist for activating autophagy under these conditions. One possibility we considered is that the second isoform of the SnRK1 catalytic subunit, KIN11 [42], can substitute for KIN10 under some conditions, including osmotic stress. Previous work has shown that the two isoforms can act antagonistically [44], suggesting that each protein has specific functions. However, a *kin10 kin11* double mutant results in lethality [42], suggesting some degree of functional overlap.

T6P inhibits SnRK1 complex activity in *Arabidopsis*, including both KIN10- and KIN11- containing complexes [45]. T6P reduces SnRK1 activity by ~80%, and even at higher concentrations (up to 4 mM) does not completely block activity [45]. Incubation of *Arabidopsis* seedlings with T6P inhibited autophagy activation in response to nutrient starvation, salt stress, oxidative stress and ER stress, consistent with the *kin10* mutant phenotype. Inhibition of SnRK1 by T6P resulted in only partial inhibition of autophagy activity in response to osmotic stress, as also seen in the *kin10* mutant. The presence of KIN11 therefore cannot account for the partial activation of autophagy observed under osmotic stress in the *kin10* mutant. We hypothesize that autophagy can be regulated by two parallel pathways during osmotic stress: one that is SnRK1-dependent and another that is SnRK1-independent.

TOR negatively regulates autophagy in many organisms, including *Arabidopsis* [17], and regulation of autophagy by AMPK can occur either through TOR or independently of TOR [36, 75]. AMPK regulates autophagy during nutrient starvation by phosphorylating RAPTOR, resulting in the inhibition of TOR and therefore activating autophagy [75]. Recent studies have shown that KIN10 can interact with RAPTOR1B *in vivo* and phosphorylate it *in vitro* [52]. We show here that activation of autophagy by increasing KIN10 activity is blocked upon activation of TOR, whereas disruption of KIN10 activity does not block the constitutive autophagy seen upon inhibition of TOR. Our results demonstrate that KIN10 acts upstream of TOR in the regulation of autophagy in *Arabidopsis* via a TOR-dependent pathway.

The activation of autophagy by ER or oxidative stress is not blocked by activation of TOR, suggesting that it occurs via a TOR-independent pathway [26]. Under ER stress conditions, autophagy is activated by the accumulation of unfolded proteins [55] and requires the unconventional splicing factor IRE1, although it is independent of IRE1’s downstream splicing target bZIP60 [12]. The signaling pathway for activation of autophagy during ER stress therefore appears to be distinct from that during nutrient stress, although it still requires SnRK1 activity. This suggests that, like AMPK and Snf1, KIN10 can also regulate autophagy through a TOR-independent pathway under specific conditions.

In conclusion, we have identified KIN10 as a positive regulator of autophagy in *Arabidopsis*. KIN10 is necessary for the activation of autophagy by many abiotic stresses, either through TOR-dependent or TOR-independent pathways, depending on the stress. In the TOR-dependent pathway, KIN10 acts upstream of TOR, probably by phosphorylating the TOR complex, to positively regulate autophagy. Further work is necessary to determine the exact role of the SnRK1 complex in the regulation of autophagy in response to osmotic stress, to clarify the mechanism by which KIN10 can regulate autophagy independently of TOR, and to identify downstream targets of TOR that contributes to the regulation of autophagy in plants.

## Materials and methods

### Plant materials and growth conditions

Wild-type (Col-0), GFP-ATG8e [14], *kin10* (SALK_127939C) [57, 58], KIN10 OX-1, KIN10 OX-2 [42], TOR overexpression [61] and *raptor1b* (SALK_078159) [18] *Arabidopsis thaliana* seeds were surface sterilized with 0.1% (v/v) Triton X-100, 33% (v/v) bleach solution for 20 minutes, rinsed 5 times with sterile water, and kept at 4°C in the dark for at least 2 days. Plants were grown on half-strength MS solid medium (Murashige-Skoog with vitamins mixture [Caisson, MSP09], 1% sucrose, 2.4 mM MES [pH 5.7], 0.6% Phytoblend agar) or in soil under long day conditions (16 h light) at 22°C.

### Stress and chemical treatments

For salt stress, 7-day-old seedlings were transferred to half-strength MS liquid medium with 160 mM NaCl for 6 hours [11]. For osmotic stress, 7-day-old seedlings were transferred to half-strength MS liquid medium with 350 mM mannitol for 6 hours [11]. For oxidative stress, 7-day-old seedlings were transferred to half-strength MS liquid medium supplemented with 10 mM hydrogen peroxide for 2 hours [14]. For ER stress treatment, 7-day-old seedlings were transferred to half-strength MS liquid medium supplemented with 2 mM dithiothreitol (DTT) for 6 hours [12]. For starvation stress, 7-day-old seedlings were transferred to half-strength MS plates lacking sucrose or nitrogen for 4 days. Plants grown on sucrose starvation plates were incubated in the dark.

For SnRK1 inhibitor treatment, 7-day old seedlings were transferred to half-strength MS liquid medium supplemented with 0.1 mM trehalose-6-phosphate (T6P) [Santa Cruz, SC216004] for 3 hours [45]. For treatment in the presence of stress, seedlings were subjected to the abiotic stress conditions as described above and T6P was added to a final concentration of 0.1 mM for the last 3 hours of the treatment. Seedlings under starvation stress were transferred after the 4 days of starvation to half-strength MS liquid medium lacking sucrose or nitrogen and supplemented with 0.1 mM T6P for 3 hours.

For SnRK1 activation, 7-day-old seedlings were transferred to half-strength MS liquid medium supplemented with 10 mM 5-aminoimidazole-4-carboxamide ribonucleoside monophosphate (AICAR) [EMD Millipore Calbiochem, 12304125MG] for 1 hour [56].

For TOR inhibitor treatment, 7-day-old seedlings were transferred to half-strength MS liquid medium supplemented with 10 μM AZD8055 (AZD) or DMSO as solvent control for 3 hours [60].

For TOR activation treatment, 7-day-old seedlings were transferred to half-strength MS liquid medium supplemented with 20 nM 1-naphthaleneacetic acid (NAA) [Sigma, N1641] or DMSO as solvent control for 6 hours.

### MDC staining and microscopy

*Arabidopsis* seedlings were stained with 0.05 mM monodansylcadaverine (MDC) [Sigma, 30432] for 10 minutes, followed by 3 brief washes with phosphate-buffered saline [53]. Seedlings were observed using a Zeiss Axioplan II compound microscope equipped with an Axio Cam HRC digital imaging system at the Iowa State University Microscopy and Nanoimaging Facility, using a X40 objective and a 4’, 6-diamidino-2-phenylindole (DAPI) filter. GFP fluorescence was imaged using a Zeiss AxioImager microscope with a X40 objective and a fluorescein isothiocyanate (FITC) filter at the Iowa State Microscopy and Nanoimaging Facility.

Confocal microscopy was performed using a Leica SP5 confocal laser scanning microscope with a X63 oil immersion objective at the Iowa State University Confocal and Multiphoton Facility. The excitation and emission wavelengths for GFP were 488 and 507 nm respectively. The excitation and emission wavelengths for MDC were 435 and 455 nm respectively.

### Transient transformation of leaf protoplasts

Leaf protoplasts were prepared from 4–6- week- old *Arabidopsis* plants [76], and 20 μg of plasmid DNA was used for each transformation. After transformation, protoplasts were incubated at room temperature overnight in darkness to allow expression, with 40 rpm orbital shaking, followed by stress and/or addition of chemicals to the protoplast suspension. Concentrations and incubation times were as described above (stress and chemical treatments section). Autophagosomes labeled with GFP-ATG8e were counted under an epifluorescence microscope. A protoplast was considered to have active autophagy if 3 or more GFP-ATG8e-labeled autophagosomes were detected [55].

For immunoblotting, protoplasts were collected by centrifugation at 300 rpm. Protein was dissolved in 3X SDS loading buffer [6% (w/v) SDS, 20% (v/v) glycerol, 125 mM Tris-HCl pH 6.8]. Proteins were separated by 12% SDS-polyacrylamide gel electrophoresis and analyzed by western blot using anti-GFP antibody [Life Technologies, A11122].

### Image analysis

Autophagosomes labeled with MDC or GFP-ATG8e in the elongation zone in the roots were manually counted, and only individual motile dots were counted as autophagosomes. GFP-ATG8e puncta in leaf protoplasts were directly counted under fluorescence microscopy.

### Generation of FLAG-KIN10

The *KIN10* cDNA was synthesized by RT-PCR from total RNA from 10-day-old seedlings grown on half strength MS plates, using gene-specific primers (forward 5’-CACCGGTACCGATTACAAGGATGACGACGATAAGATGGATGGATCAGGCACAGG-3’, reverse 5’-AACACCGAGCTCTCAGAGGACTCGGAGCTGAG-3’). The forward primer also encoded the FLAG tag for detection of expression. The cDNA was ligated into the MCS11 binary vector using KpnI and SacI restriction sites. The final construct was verified by enzymatic digestion and sequencing.

## Supporting information

S1 Fig Transient expression of GFP-ATG8e in KIN10 overexpression lines. (A) The autophagosome marker GFP-ATG8e was transiently expressed in leaf protoplasts from the indicated genotypes and visualized by confocal microscopy. KIN10 overexpression lines have increased autophagy activity when compared to WT. White arrows point to autophagosomes. Scale bar = 10 μm. (B) Immunoblotting of protein extracts from protoplasts as in (A) using antibodies against GFP. Ponceau S stain was used as loading control. All samples show approximately equal expression of GFP-ATG8e.
(PDF)

S2 Fig Transient expression of GFP-ATG8e in *kin10* mutant protoplasts. (A) The autophagosome marker GFP-ATG8e was transiently expressed in leaf protoplasts from the indicated genotypes and visualized by confocal microscopy. After inducing ER stress as a representative stress with 2 mM DTT, the *kin10* mutant fails to activate autophagy when compared to WT. White arrows point to autophagosomes. Scale bar = 10 μm. (B) Immunoblotting of protein extracts from protoplasts as in (A) using antibodies against GFP. Ponceau S stain was used as loading control. All samples show approximately equal expression of GFP-ATG8e.
(PDF)

S3 Fig Complementation of the *kin10* mutant. (A) The autophagosome marker GFP-ATG8e was transiently expressed in leaf protoplasts from the indicated genotypes and visualized by confocal microscopy. After inducing ER stress as a representative stress with 2 mM DTT, *kin10*:FLAG-KIN10 shows induction of autophagy as in WT, while *kin10* mutant fails to induce autophagy. White arrows point to autophagosomes. Scale bar = 10 μm. (B) Immunoblotting of protein extracts from protoplasts as in (A) using antibodies against GFP. Ponceau S stain was used as loading control. All samples show approximately equal expression of GFP-ATG8e.
(PDF)
