# Supplementary material for: SnRK1 activates autophagy via the TOR signaling pathway in Arabidopsis thaliana
Source: PLoS One. 2017 Aug 4;12(8):e0182591. doi: 10.1371/journal.pone.0182591 (PMC5544219; doi:10.1371/journal.pone.0182591)
Supplement: S1 Fig — (A) The autophagosome marker GFP-ATG8e was transiently expressed in leaf protoplasts from the indicated genotypes and visualized by confocal microscopy. KIN10 overexpression lines have increased autophagy activity when compared to WT. White arrows point to autophagosomes. Scale bar = 10 μm. (B) Immunoblotting of protein extracts from protoplasts as in (A) using antibodies against GFP. Ponceau S stain was used as loading control. All samples show approximately equal expression of GFP-ATG8e. (PDF) [file pone.0182591.s001.pdf]

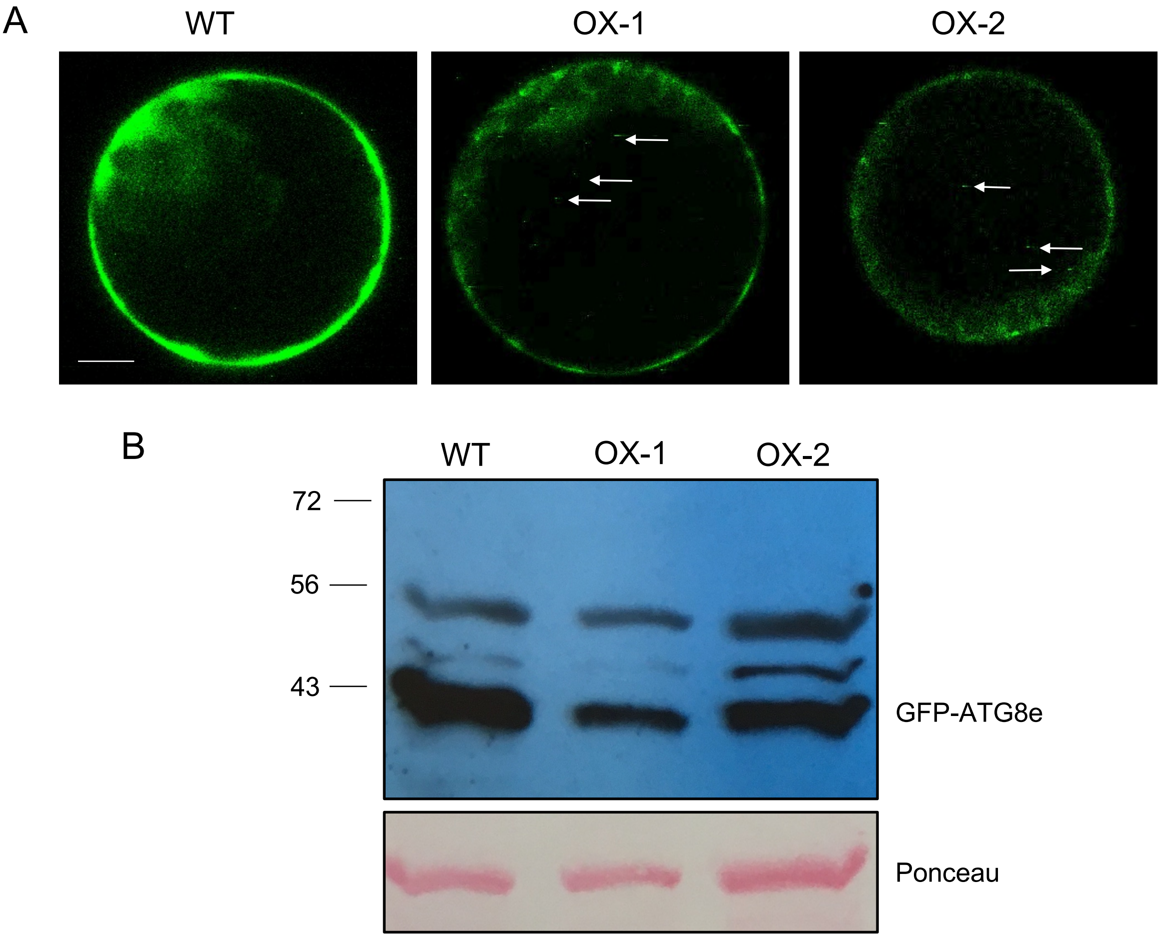

S1 Fig. Transient expression of GFP-ATG8e in KIN10 overexpression lines

(A) The autophagosome marker GFP-ATG8e was transiently expressed in leaf protoplasts from the indicated genotypes and visualized by confocal microscopy. KIN10 overexpression lines have increased autophagy activity when compared to WT. White arrows point to autophagosomes. Scale bar = 10 μm. (B) Immunoblotting of protein extracts from protoplasts as in (A) using antibodies against GFP. Ponceau S stain was used as loading control. All samples show approximately equal expression of GFP-ATG8e.
