# Supplementary material for: SnRK1 activates autophagy via the TOR signaling pathway in Arabidopsis thaliana
Source: PLoS One. 2017 Aug 4;12(8):e0182591. doi: 10.1371/journal.pone.0182591 (PMC5544219; doi:10.1371/journal.pone.0182591)
Supplement: S2 Fig — (A) The autophagosome marker GFP-ATG8e was transiently expressed in leaf protoplasts from the indicated genotypes and visualized by confocal microscopy. After inducing ER stress as a representative stress with 2 mM DTT, the kin10 mutant fails to activate autophagy when compared to WT. White arrows point to autophagosomes. Scale bar = 10 μm. (B) Immunoblotting of protein extracts from protoplasts as in (A) using antibodies against GFP. Ponceau S stain was used as loading control. All samples show approximately equal expression of GFP-ATG8e. (PDF) [file pone.0182591.s002.pdf]

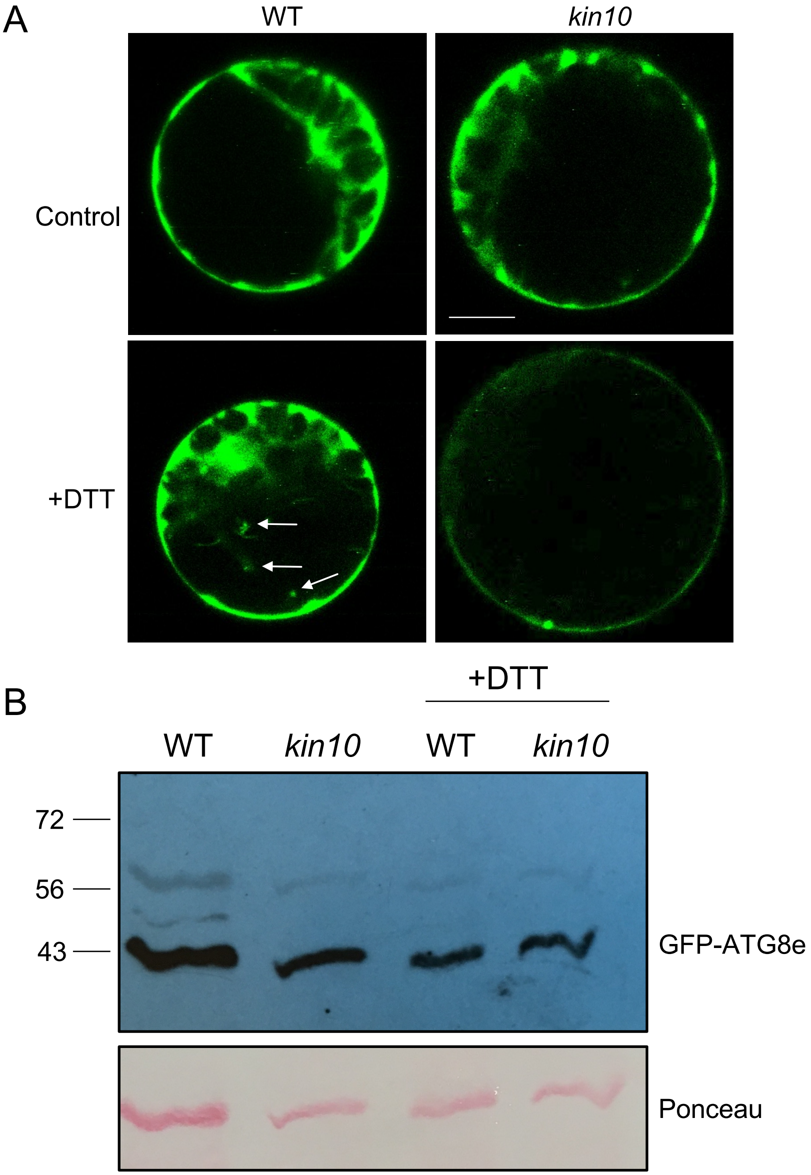

S2 Fig. Transient expression of GFP-ATG8e in *kin10* mutant protoplasts

(A) The autophagosome marker GFP-ATG8e was transiently expressed in leaf protoplasts from the indicated genotypes and visualized by confocal microscopy. After inducing ER stress as a representative stress with 2 mM DTT, the *kin10* mutant fails to activate autophagy when compared to WT. White arrows point to autophagosomes. Scale bar = 10 μm. (B) Immunoblotting of protein extracts from protoplasts as in (A) using antibodies against GFP. Ponceau S stain was used as loading control. All samples show approximately equal expression of GFP-ATG8e.
