# Supplementary material for: SnRK1 activates autophagy via the TOR signaling pathway in Arabidopsis thaliana
Source: PLoS One. 2017 Aug 4;12(8):e0182591. doi: 10.1371/journal.pone.0182591 (PMC5544219; doi:10.1371/journal.pone.0182591)
Supplement: S3 Fig — (A) The autophagosome marker GFP-ATG8e was transiently expressed in leaf protoplasts from the indicated genotypes and visualized by confocal microscopy. After inducing ER stress as a representative stress with 2 mM DTT, kin10:FLAG-KIN10 shows induction of autophagy as in WT, while kin10 mutant fails to induce autophagy. White arrows point to autophagosomes. Scale bar = 10 μm. (B) Immunoblotting of protein extracts from protoplasts as in (A) using antibodies against GFP. Ponceau S stain was used as loading control. All samples show approximately equal expression of GFP-ATG8e. (PDF) [file pone.0182591.s003.pdf]

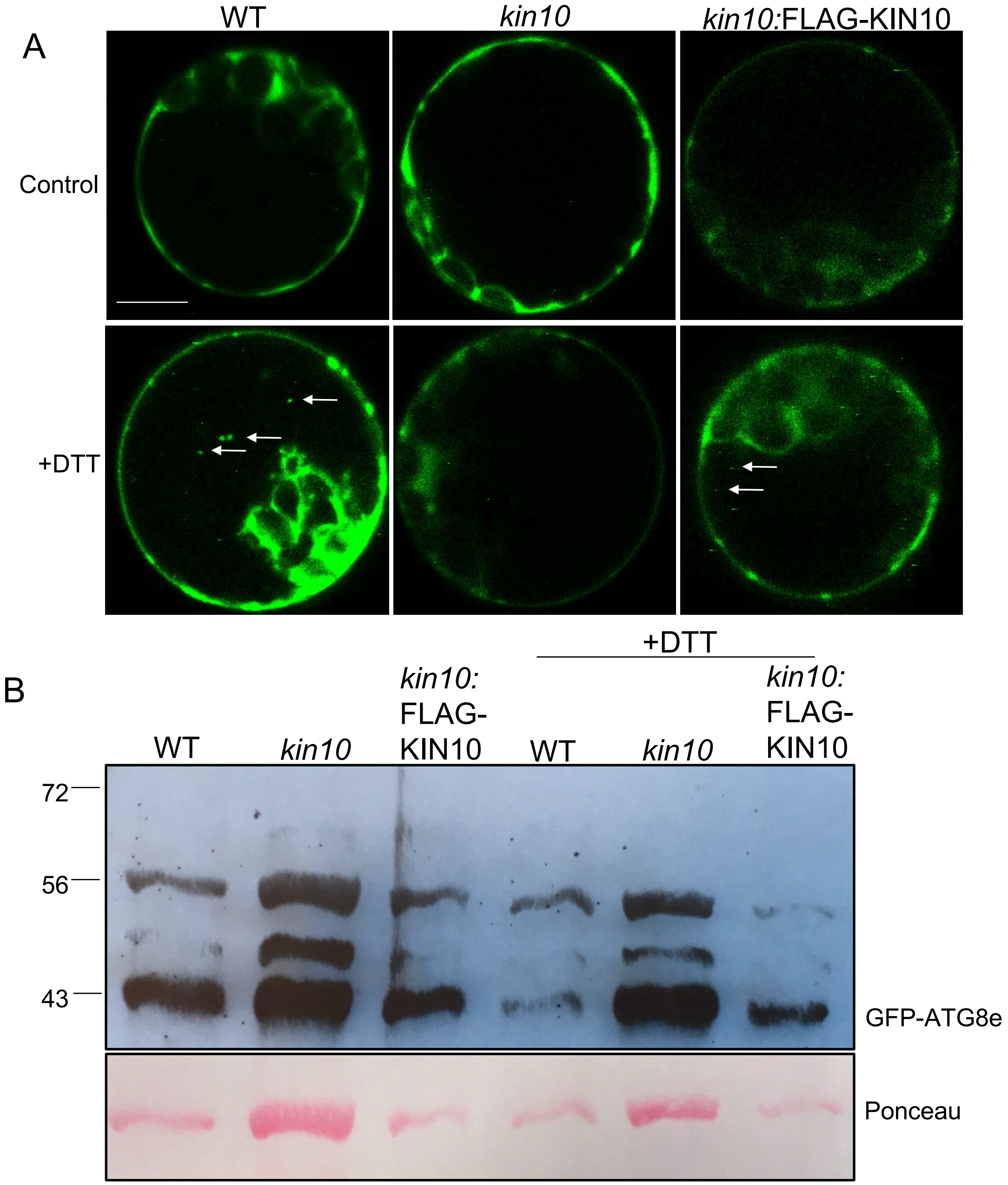

S3 Fig. Complementation of the *kin10* mutant

(A) The autophagosome marker GFP-ATG8e was transiently expressed in leaf protoplasts from the indicated genotypes and visualized by confocal microscopy. After inducing ER stress as a representative stress with 2 mM DTT, *kin10*:FLAG-KIN10 shows induction of autophagy as in WT, while *kin10* mutant fails to induce autophagy. White arrows point to autophagosomes. Scale bar = 10 μm. (B) Immunoblotting of protein extracts from protoplasts as in (A) using antibodies against GFP. Ponceau S stain was used as loading control. All samples show approximately equal expression of GFP-ATG8e.
